# Chromatic analysis of orthodontic resin bonding agents exposed to different antiseptic mouthrinses

**DOI:** 10.1590/2177-6709.26.2.e211955.oar

**Published:** 2021-05-17

**Authors:** Alana Dantas MOREIRA, Jamille Barros FERREIRA, Claudia Trindade MATTOS, Mariana MARQUEZAN, Mônica Tirre de Souza ARAÚJO, Eduardo Franzotti SANT’ANNA

**Affiliations:** 1 Universidade Federal do Rio de Janeiro, Departamento de Odontopediatria e Ortodontia (Rio de Janeiro/RJ, Brazil).; 2 Universidade Federal Fluminense, Departamento de Ortodontia (Niterói/RJ, Brazil).; 3 Universidade Federal de Santa Maria, Departamento de Estomatologia (Santa Maria/RS, Brazil).

**Keywords:** Orthodontics. Resins, synthetic. Mouthwashes

## Abstract

**Objective:**

To assess the color of different orthodontic resin bonding agents exposed to three antiseptic mouthrinses for a prolonged time interval (10-year aging simulation).

**Methods:**

160 specimens were distributed into four groups, according to the orthodontic resin bond agent (Concise, Transbond XT, Transbond Plus Color Change, and Natural Ortho). Each group was exposed to different antiseptic mouthrinses: alcohol-based (Listerine®), alcohol-free (Oral-B®), chlorhexidine (Periogard®) and distilled water as the control. Specimens were submitted to two cycles of staining and artificial aging. Color was evaluated by means of a digital spectrophotometer at the beginning of the experiment and after every cycle. The system used to assess color changes was the CIE L*a*b*. Data was analyzed using the ANOVA and Tukey *post-hoc* test.

**Results:**

After simulation of 10 years of aging, Transbond XT and Natural Ortho composites presented no statistically significant differences in ∆E when exposed to different mouthrinses. The Concise composite specimens exposed to alcohol-free mouthrinse presented a significant difference when compared with specimens from the same group exposed to other antiseptic mouthrinses. Transbond Plus Color Change specimens exposed to chlorhexidine mouthrinse and to alcohol-containing mouthrinse presented a significant difference when compared with the specimens from the group exposed to water and alcohol-free antiseptic.

**Conclusion:**

All orthodontic resin bonding agents tested presented clinically perceptible color changes when exposed to at least one of the mouthrinses, except for the Natural Ortho composite. The Concise composite exposed to the alcohol-free solution was the resin that presented the highest color change values.

## RESUMO

**Objetivo:**

Avaliar a cor de diferentes resinas ortodônticas expostas a três enxaguantes bucais, por um intervalo de tempo prolongado (simulação de 10 anos de envelhecimento).

**Métodos:**

160 espécimes foram distribuídos em quatro grupos, de acordo com a resina de colagem ortodôntica (Concise, Transbond XT, Transbond Plus Color Change e Natural Ortho). Cada grupo foi exposto a diferentes tipos de enxaguantes bucais: à base de álcool (Listerine®), isento de álcool (Oral-B®), clorexidina (Periogard®) ou água destilada, como controle. Os espécimes foram submetidos a dois ciclos de manchamento e envelhecimento artificial. A cor foi avaliada por meio de um espectrofotômetro digital no início do experimento e após cada ciclo. O sistema utilizado para avaliar as alterações de cor foi o CIE L*a*b*. Os dados foram analisados utilizando-se o teste ANOVA e o teste *post-hoc* de Tukey.

**Resultados:**

Após a simulação de 10 anos de envelhecimento, as resinas Transbond XT e Natural Ortho não apresentaram diferenças estatisticamente significativas no ∆E, quando expostas a diferentes enxaguantes bucais. Os espécimes do grupo Concise expostos ao enxaguante bucal sem álcool apresentaram diferença significativa quando comparados aos espécimes do mesmo grupo expostos aos outros enxaguantes bucais. Os espécimes do grupo Transbond Plus Color Change expostos à clorexidina e ao enxaguante bucal à base de álcool apresentaram diferença significativa quando comparados aos espécimes dos grupos expostos à água e ao enxaguante bucal sem álcool.

**Conclusão:**

Todas as resinas ortodônticas avaliadas apresentaram alterações de cor clinicamente perceptíveis quando expostas a pelo menos um dos enxaguantes bucais, com exceção da resina ortodôntica Natural Ortho. A resina Concise exposta à solução isenta de álcool foi a que apresentou os maiores valores de alteração de cor.

## INTRODUCTION

The constant evolution of techniques, materials and concepts in Dentistry demands that dentists keep up to date with these innovations. This is especially important with regard to new trends in esthetics, considering factors that include tooth color, shape and alignment, as well as facial expressions and gingival appearance.^1^ A more demanding orthodontic patient is also generally concerned with a possible tooth color change during and after treatment. Depending on the frequency and period of exposure to agents that affect color, some environmental factors may cause changes in esthetics during treatment.^2^

The main purpose of using antiseptic mouthrinses is to control the development and progression of periodontal disease and caries. However, their frequent use may lead to adverse effects on teeth and oral tissues,^3^ ^,^ ^4^ ^,^ ^5^ including chromatic changes. As a result, several studies have analyzed the color stability of dental materials exposed to alcohol-containing, alcohol-free and chlorhexidine antiseptic mouthrinses.^3^ ^,^ ^5^ ^-^ ^8^

Alcohol and chlorhexidine have unique characteristics that have contributed to their addition to mouthrinse solutions and both have antiseptic properties. Alcohol helps the breakdown or dissolution of active principles and preserves the components of the formula. Chlorhexidine is capable of denaturing the components of biofilm. Among their disadvantages and side effects, alcohol may be responsible for lesions in oral tissues and softening of resin composites;^9^ ^,^ ^10^ and chlorhexidine may be associated with changes in sensitivity, superficial peeling of the oral mucosa, calculus formation and change in color of the tongue and teeth, resulting from the precipitation of dietary pigments.^4^ ^,^ ^11^

The lack of color stability of orthodontic resin bonding agents is a main source of tooth darkening or staining. Acid conditioning on dental enamel performed before the procedure of bonding orthodontic fixed appliances to the teeth results in an increase in microporosities on their surface. When these microporosities are filled with resin, tags (resin extension within the dental enamel) are created, and the depth and thickness of these tags is highly variable, with their mean size ranging from 11.8 µm to 18.9 µm. Many tags may reach a depth of 89 µm to 100 µm^12^. These tags are intended to provide retention between the orthodontic bonding agent and the tooth, and they may remain in the dental enamel permanently. Nevertheless, the aging process in addition to pigments present in a person’s diet, and chemical products used in the oral cavity may alter the color of these composites, leading to poor esthetics.^13^

Additionally, it is commonplace for a certain amount of excess resin bonding agent flash to remain on the bracket edges; between the bracket and the enamel, during bracket bonding.^14^ Armstrong et al.^14^ observed that even the addition of a color change feature in the bonding agent does not guarantee a reduction in the amount of excessive resin bonding agent accumulating around orthodontic brackets. Therefore, the color change in these excessive orthodontic bonding agents could be esthetically important during orthodontic treatment, particularly in the anterior teeth.

Thus, the aim of this study was to perform an *in vitro* chromatic analysis of orthodontic resin bonding agents exposed to different antiseptic mouthrinses for a prolonged time interval.

## MATERIAL AND METHODS

In this study, 160 5mm-diameter and 2mm-high disc-shaped specimens were made from four different orthodontic resin bonding agents and divided into four groups, according to the orthodontic resin. All specimens obtained were immersed in distilled water at a temperature of 37^o^C for 24 hours, in order to ensure complete polymerization.^6^

Each group consisted of 40 specimens that were made from each of the following bonding agents: 1) Concise composite chemical cure resin bonding agent (3M Unitek, Monrovia, USA), 2) Transbond XT light cure resin bonding agent (3M Unitek, Monrovia, USA), 3) Transbond Plus Color Change light cure resin bonding agent (3M Unitek, Monrovia, USA) and 4) Natural Ortho light cure orthodontic resin bonding agent (DFL, Rio de Janeiro, Brazil). Ten specimens of each group were exposed to different mouthrinses: an alcohol-containing (21.6%) mouthrinse (Listerine^®^, Tartar Control, Johnson & Johnson, São Paulo, Brazil); an alcohol-free antiseptic (cetylpyridinium chloride) mouthrinse (Oral-B^®^, Mint flavor, Procter & Gamble, São Paulo, Brazil); a chlorhexidine (0.06%) mouthrinse (Periogard^®^, Colgate-Palmolive, São Paulo, Brazil); and distilled water served as control.^3^ All mouthrinses tested had a blue color.

All specimens, immersed in their respective solutions, were submitted to two cycles of staining and artificial aging in an aging chamber with ultraviolet light (wavelength of 254 nm), under heat (45^o^C) and 65% relative humidity (according to ADA Standard n. 27), for 24 hours. Each cycle corresponded to 5 years of aging in the mouth. During the interval between cycles and at the end of the experiment, the specimens were immersed in distilled water. 

The color of specimens was assessed with a portable digital spectrophotometer^3^ ^,^ ^6^ Vita Easyshade^®^ Compact (VITA Zahnfabrik H. Rauter GmbH Co. KG, Bad Säckingen, Germany - Model DEASYC220) at two time intervals: initial (T_0_) - before their immersion in the respective solutions; and after the second cycle (T_1_). The measurements were made in the same environment by a single, previously calibrated operator. Three color measurements were taken of each specimen at each time interval, and the mean of these values was considered.^3^

The Commission Internationale de L’Eclairage (CIE L*a*b*)^15^ ^,^ ^16^ defined a system of color reading, where L* represents the luminosity axis (from black to white). The chroma is described by two variables: a* and b*; a* represents the green-red axis (-a = green; +a = red), and b* represents the blue-yellow axis (-b = blue; +b = yellow). Thus, the calculation of total color change (∆E*ab) was possible by using the following formula: ΔE*ab = [(ΔL)^2^+ (Δa)^2^+ (Δb)^2^]^1/2^. The values of L*, b* and ∆E* were compared.^16^

The results obtained were analyzed using the Kolmogorov-Smirnov normality test. Analysis of variance (ANOVA) and the Tukey *post-hoc* tests were used to identify differences between groups in each period. The paired-samples *t*-test was used to identify differences related to mouthrinses in each group between time intervals, compared to distilled water.

## RESULTS

All four resins showed some color change when immersed in any of the tested solutions, however, the resins presented different behavior when exposed to the individual solutions. Considering ∆E values, Concise resin showed a more significant color change only when immersed in Oral-B rinse. Transbond Plus Color Change showed higher color change values when immersed in Oral-B and Periogard. Transbond XT and Natural Ortho, on the other hand, showed no statistically significant difference, regardless of the solutions in which they were immersed (Table 1). 

**Table 1:** Descriptive statistics and comparisons of ∆E values among different resins and among mouthrinses after the two cycles of staining and artificial aging (T1).

	Concise	Transbond XT	Transbond Plus Color Change	Natural Ortho
	Mean (SD)	Mean (SD)	Mean (SD)	Mean (SD)
Distilled water	2.55 (0.93)^ABa^	3.25 (1.72)^Ba^	2.50 (1.04)^ABa^	1.35 (1.38)^Aa^
Listerine^®^	3.31 (1.31)^ABa^	3.64 (1.35)^Ba^	2.68 (0.96) ^ABa^	1.99 (0.92)^Aa^
Oral-B^®^	6.32 (2.54)^Ab^	3.64 (0.97)^Ba^	2.92 (1.09)^Bab^	2.53 (0.92)^Ba^
Periogard^®^	2.29 (0.83)^Aa^	2.59 (1.13)^Aa^	3.86 (0.60)^Bb^	1.55 (0.98)^Aa^

^A,B^ Comparison among resins (each line express a different test).

^a,b^ Comparison among mouthrinses (each column express a different test).

Different letters mean statistically significant difference (p<0.05).

When resins were immersed in water (control), only Transbond XT showed a significant change in ∆E. When immersed in Listerine, the most significant color change was observed for Transbond XT, and Natural Ortho showed the least significant change. Oral-B caused higher color change values in Concise, while Periogard was the strongest color change mouthrinse on Transbond Plus Color Change (Table 1). 

The comparison between initial and final L values found for the Concise specimens showed a statistically significant difference among all mouthrinses studied. Transbond XT and Transbond Plus Color Change specimens exposed to the Listerine^®^ solution and to Periogard^®^ showed statistically significant difference between the initial and final L values.. The comparison between initial and final L values found for the Natural Ortho specimens showed a statistically significant difference when exposed to the Listerine^®^ solution (Table 2).

**Table 2:** Descriptive statistics (mean and standard deviation) and comparisons of L values between mouthrinses in each group after the two cycles of staining and artificial aging.

Solutions	Concise		Transbond XT		Transbond Plus Color Change		Natural Ortho	
	T_0_	T_1_	T_0_	T_1_	T_0_	T_1_	T_0_	T_1_
Distilled water	90.27 (0.74)^a^	88.33 (1.03)^b^	86.55 (0.15)^a^	86.32 (0.34)^a^	93.77 (1.01)^a^	93.32 (0.68)^a^	95.72 (0.55)^a^	95.46 (0.54)^a^
Listerine^®^	90.22 (1.06)^a^	89.38 (1.31)^b^	86.74 (0.63)^a^	85.98 (0.55)^b^	94.24 (0.93)^a^	93.85 (1.23)^b^	95.60 (0.65)^a^	95.05 (0.60)^b^
Oral-B^®^	90.68 (1.17)^a^	87.57 (2.40)^b^	86.82 (0.41)^a^	86.75 (0.68)^a^	93.21 (0.56)^a^	93.31 (0.83)^a^	95.91 (0.73)^a^	95.56 (0.76)^a^
Periogard ^®^	86.69 (0.91)^a^	88.91 (0.67)^b^	86.62 (0.39)^a^	86.25 (0.35)^c^	94.60 (1.35)^a^	93.71 (1.13)^b^	95.71 (0.94)^a^	95.72 (0.88)^a^

^a,b^ Comparison among the mouthrinses (each column express a different test).

Different letters mean statistically significant difference (p<0.05).

Comparison between the initial and final b* values of the Concise specimens showed a statistically significant difference for all the mouthrinses studied, except for Oral-B^®^ solution. Transbond XT, Transbond Plus Color Change and Natural Ortho specimens showed a statistically significant difference between the initial and final b* values when immersed in any of the mouthrinses studied and distilled water (Table 3).

**Table 3:** Descriptive statistics (mean and standard deviation) and comparisons of b* values between mouthrinses in each group after the two cycles of staining and artificial aging.

Solutions	Concise		Transbond XT		Transbond Plus Color Change		Natural Ortho	
	T_0_	T_1_	T_0_	T_1_	T_0_	T_1_	T_0_	T_1_
Distilled water	25.48 (0.64)^a^	26.73 (1.35)^b^	16.04 (2.27)^a^	13.60 (1.42)^b^	19.14 (1.32)^a^	16.99 (0.71)^b^	16.51 (1.02)^a^	15.43 (0.91)^b^
Listerine^®^	24.89 (1.25)^a^	27.75 (1.33)^b^	15.12 (1.66)^a^	11,68 (0,56)^b^	17.97 (0.98)^a^	15.77 (1.13)^c^	16.14 (1.11)^a^	14.78 (0.96)^b^
Oral-B^®^	23.72 (1.10)^a^	23.61 (1.85)^a^	14.40 (1.01)^a^	10.82 (0.64)^b^	17.86 (0.69)^a^	15.68 (1.34)^c^	15.97 (1.07)^a^	13.54 (0.66)^c^
Periogard ^®^	24.60 (1.01)^a^	25.86 (0.81)^c^	14.70 (1.52)^a^	12.21 (1.10)^b^	17.96 (0.74)^a^	14.62 (0.49)^b^	15.78 (1.24)^a^	14.43 (0.82)^b^

^a,b^ Comparison among the mouthrinses (each column express a different test).

Different letters mean statistically significant difference (p<0.05).

## DISCUSSION

Visual color perception is essentially a subjective matter, which may be physiologically and psychologically influenced, and may be different for each researcher willing to assess color changes. Some of the factors that may influence visual color assessment and contribute to this subjectivity are: the distance between the object and the observer, the color of the light used for illumination, the metamerism phenomenon, fatigue and aging of the object, and even the emotional state of the observer.^17^ In this context, the use of a spectrophotometer eliminates the errors of subjective color assessment.

The ∆E values are frequently used to assess color change between two time intervals, although they do not reveal specifically where this change lies. Some authors consider a ∆E value of 3.3 as the threshold, after which the color change becomes visually perceptible,^18^ ^,^ ^19^ while other authors have adopted the value of 3.7 as the threshold.^20^ ^,^ ^21^ The ∆E limit value considered in this study was 3.3.

Concise specimens exposed to the Oral-B^®^ solution presented the highest ∆E values (6.32) among all specimens and solutions studied. The specimens of the same composite exposed to the Listerine^®^ solution also showed a clinically perceptible color change (∆E = 3.31), but to a lower degree. Transbond XT specimens showed an equally perceptible change (∆E = 3.64) when submitted to both Oral-B^®^ and Listerine^®^ solutions. Transbond Plus Color Change specimens, however, only presented ∆E values (3.86) that characterized a clinically perceptible color change when they were exposed to the Periogard^®^ solution. Natural Ortho specimens presented no visually perceptible color changes, according to the threshold of ∆E considered in this study (3.3).^18^ ^,^ ^19^

Luminosity is the most important factor in determining color, as colors with low luminosity values appear to be darker. The assessment of L* values allowed the analysis of whether the studied resin bonding agents became lighter or darker. When the L* values rose, this meant that the luminosity increased, and the object became lighter. When the L* values decreased, this meant that there was a reduction in the luminosity and the object became darker. Changes in the L* values are the most significant parameter in color change, as the human eye may detect these changes more easily than changes in other parameters, such as the a* and b* values. Any change in L* values below 2.0 is not clinically visible.^21^

All bonding agents assessed in this study became darker or showed no difference in luminosity after the two cycles of staining and artificial aging, when immersed in distilled water and when they were exposed to different mouthrinses. The only exception was the Transbond Plus Color Change specimens exposed to the Oral-B^®^ solution, which showed a discrete increase in luminosity that was not statistically significant.

Since b* values may represent a yellow (positive values) or a blue (negative values) hue, analysis of the b* values in this study showed that Transbond XT, Transbond Plus Color Change and Natural Ortho specimens presented a color change tending towards a blue hue, confirmed by the reduction in b* values after the two cycles of staining and artificial aging. However, Concise composite specimens presented a decrease in b* values only when exposed to the Oral-B^®^ solution. The specimens exposed to Listerine^®^ and Periogard^®^ solutions, as well as to distilled water, showed an increase in b* values, indicating a color change tending towards a yellow hue by the end of the experiment.

In this study, the effect of an alcohol-containing mouthrinse, an alcohol-free antiseptic mouthrinse and a chlorhexidine mouthrinse on color stability of different orthodontic resin bonding agents was assessed. Villalta et al.^22^ noted that a low pH and a high alcohol concentration in solutions may affect the surface integrity and stain dental composite resins. In this study, two orthodontic bonding agents (Transbond XT and Concise) submitted to the alcohol-containing solution Listerine^®^ presented a perceptible color change, and two (Transbond Plus Color Change and Natural Ortho) presented an imperceptible color change. Chlorhexidine may denature the components of biofilm, accelerating the formation of pigmented sulfides and precipitating pigments from diet.^9^ In this *in vitro* study, the use of food or diet was not included, so that the lack of staining in specimens submitted to the chlorhexidine solution Periogard^®^ could be justified. Whereas, one bonding agent (Transbond Plus Color Change) presented a clinically perceptible color change when exposed to the chlorhexidine solution Periogard^®^.

According to Eliades et al,^13^ one of the sources of exogenous discoloration of polymeric materials may be the superficial absorption of color pigmentation from colored mouthrinses, even though the discoloration of these materials may originate from a wide array of exogenous or endogenous sources. The effect of antiseptic solutions on composite color change may be material-dependent and the resin susceptibility to staining may be attributed to its matrix. Some authors have shown that the type of material played a significant role in resistance to staining.^3^ Likewise, in this study, different types of orthodontic resin bonding agents showed different behaviors when exposed to antiseptic mouthrinses and submitted to two cycles of artificial aging and staining. 

Clinicians should thus be aware of the properties of the orthodontic resin bonding agents they use, in order to indicate a more compatible mouthrinse that affects it the least. Remnants of orthodontic resin bonding agents around metal orthodontic brackets, which become stained, may impair esthetics during orthodontic treatment. In addition, this staining may become even more evident in the remaining orthodontic resin bonding agents located around translucent esthetic brackets. After orthodontic treatment and bracket debonding, resin tags remain on enamel surface, so the esthetic appearance and color stability of the resin continue to be cause for concern. Therefore, to enable clinicians to maintain esthetics during and after orthodontic treatment, it is essential for them to know which orthodontic resin bonding agents are more likely to be stained with certain mouthrinses.

The results of this study showed that antiseptic mouthrinses may cause chromatic changes in orthodontic resin bonding agents. However, in general, the specific presence of alcohol or chlorhexidine in these solutions did not seem to trigger these color changes.

*In vivo* studies are necessary to observe the effect of antiseptic mouthrinses on orthodontic resin bonding agents under clinical conditions, as some factors such as saliva, biofilm and diet may not be adequately reproduced *in vitro* and may influence the physical and esthetic properties of resins.

## CONCLUSION

Orthodontic resin bonding agents tested presented clinically perceptible color changes when exposed to at least one of the tested mouthrinses, except for the Natural Ortho bonding agent, which showed no visually perceptible color changes.

The Concise bonding agent exposed to the alcohol-free solution was the resin that showed the highest color change values, which differed statistically from that of the other resins observed in the study.
